# Analysis of Behavior and Trafficking of Dendritic Cells within the Brain during Toxoplasmic Encephalitis

**DOI:** 10.1371/journal.ppat.1002246

**Published:** 2011-09-15

**Authors:** Beena John, Brendon Ricart, Elia D. Tait Wojno, Tajie H. Harris, Louise M. Randall, David A. Christian, Beth Gregg, Daniel Manzoni De Almeida, Wolfgang Weninger, Daniel A. Hammer, Christopher A. Hunter

**Affiliations:** 1 Department of Pathobiology, School of Veterinary Medicine, University of Pennsylvania, Philadelphia, Pennsylvania, United States of America; 2 Chemical and Biomolecular Engineering, University of Pennsylvania, Philadelphia, Pennsylvania, United States of America; 3 The Centenary Institute for Cancer Medicine and Cell Biology, Newtown, Australia; Washington University School of Medicine, United States of America

## Abstract

Under normal conditions the immune system has limited access to the brain; however, during toxoplasmic encephalitis (TE), large numbers of T cells and APCs accumulate within this site. A combination of real time imaging, transgenic reporter mice, and recombinant parasites allowed a comprehensive analysis of CD11c^+^ cells during TE. These studies reveal that the CNS CD11c^+^ cells consist of a mixture of microglia and dendritic cells (DCs) with distinct behavior associated with their ability to interact with parasites or effector T cells. The CNS DCs upregulated several chemokine receptors during TE, but none of these individual receptors tested was required for migration of DCs into the brain. However, this process was pertussis toxin sensitive and dependent on the integrin LFA-1, suggesting that the synergistic effect of signaling through multiple chemokine receptors, possibly leading to changes in the affinity of LFA-1, is involved in the recruitment/retention of DCs to the CNS and thus provides new insights into how the immune system accesses this unique site.

## Author Summary

Toxoplasmic encephalitis (TE), caused by the protozoan parasite *Toxoplasma gondii,* can be potentially life threatening especially in immuno-compromised individuals. Immune cells including dendritic cells have been shown to accumulate in the brain during chronic toxoplasmosis; however, little is known about their function, their behavior *in vivo*, and the mechanisms by which they migrate into the brain. In the present studies, we utilize a combination of real time imaging, transgenic reporter mice, and recombinant parasites to reveal the distinct behavior and morphologies of dendritic cells within the brain and their ability to interact with parasites and effector T cells during TE. The CNS DCs were also found to exhibit a unique chemokine receptor expression pattern during infection, and the migration of DCs into the brain was mediated through a pertussis toxin (which blocks signaling downstream of several chemokine receptors) sensitive process and dependent on the integrin LFA-1. There is currently a poor understanding of the events that lead to DC recruitment to the CNS during inflammation in general, and our studies provide new insights into the mechanisms by which antigen-presenting cells gain access to the brain during infection.

## Introduction

Resistance to the parasite *Toxoplasma gondii* is characterized by an acute phase of infection associated with activation of innate immune cells such as neutrophils, dendritic cells (DCs), and macrophages that produce IL-12, which promotes NK and T cell production of IFN-γ [1], [2] These events lead to the early control of parasite replication and the transition into the chronic phase of infection where the parasite persists as tissue cysts, most notably within the CNS [3]–[5]. While the immune privileged nature of the brain may contribute to the ability of *T. gondii* to persist, several studies have highlighted that the inflamed brain (during CNS infections and during EAE) is an immunologically active site where activated T cells are constantly surveying their environment [6]–[9]. CD4^+^ and CD8^+^ T cells contribute to the local control of *T. gondii* in the CNS but much less is known about the contribution of various APC populations within the brain to T cell mediated resistance to this infection [10]–[12].

DCs are regarded as the most potent APCs responsible for initiation of immune responses and have a critical role in promoting resistance to *T. gondii* [13]–[16]. Highly activated DCs are also present within the brain during TE [17], [18] and similar accumulation of DCs within the CNS has been reported in other models of neuro-inflammation [19], [20]. While the DCs that are found in the CNS under normal conditions (primarily in the meninges and choroid plexus) have been proposed to be tolerogenic [21]–[23], the DCs present during neuro-inflammation have been ascribed numerous functions. During EAE, DCs isolated at the onset of disease were found to prime inflammatory T cells, while the DCs isolated at the peak of disease were relatively poor APCs and supported the development of regulatory T cells [19], [24], [25]. Other studies indicate that CNS DCs can function as efficient APCs and may serve to amplify intra-cerebral T cell responses [17], [26]. Regardless, during TE, the behavior and function of DCs within the CNS remains unclear.

Multiple mechanisms have been proposed to account for the accumulation of DCs within the CNS during neuroinflammation. Some studies indicate that microglia can differentiate into DCs during inflammation [18], [27], while others have proposed that DCs in the brain arise from peripheral haematopoietic cells [19], [28]. During TE, the precise origin of the CNS DCs is unclear: whether they are a local population that is constantly renewed or they are recruited from the peripheral circulation has not been addressed. Furthermore, while recent studies have highlighted the molecular signals required for the migration of DCs within secondary lymphoid compartments [29], much less is known about the migratory patterns of DCs into peripheral sites such as the inflamed brain and the signals required for their retention within these sites.

To better understand the phenotype and function of the CNS resident DCs during TE, a combination of imaging techniques, transgenic reporter mice (CD11c^YFP^ and OT1^GFP^) and recombinant parasites expressing ovalbumin and fluorescent tags (td Tomato) were used. These studies revealed that during TE, the CD11c^+^ cells form an extensive network consisting of microglia and DCs. *In vitro* antigen presentation studies and real time imaging suggest that the CNS resident CD11c^+^ cells can function as efficient APCs. These studies also showed that CD11c^+^ cells isolated from the brain expressed higher levels of the chemokine receptors CCR7, CCR5, CXCR3, CX_3_CR1 compared to their splenic counterparts, suggesting a possible role for these receptors in the migration and retention of these cells within the brain. However, the loss of any individual chemokine receptor (tested) did not affect the ability of DCs to migrate into the brain, in an adoptive transfer model. Nevertheless, this process was pertussis toxin sensitive (indicating a role for Gα_i_ coupled chemokine receptors) and LFA-1 dependent. These studies point towards a model in which the synergistic effect of signaling through multiple chemokine receptors, potentially leads to a change in the affinity of the integrin LFA-1 on the surface of DCs and controls their migration and retention within the CNS during TE.

## Results

### Characterization of the CD11c^+^ cells in the brain during chronic infection

**Figure 1:**
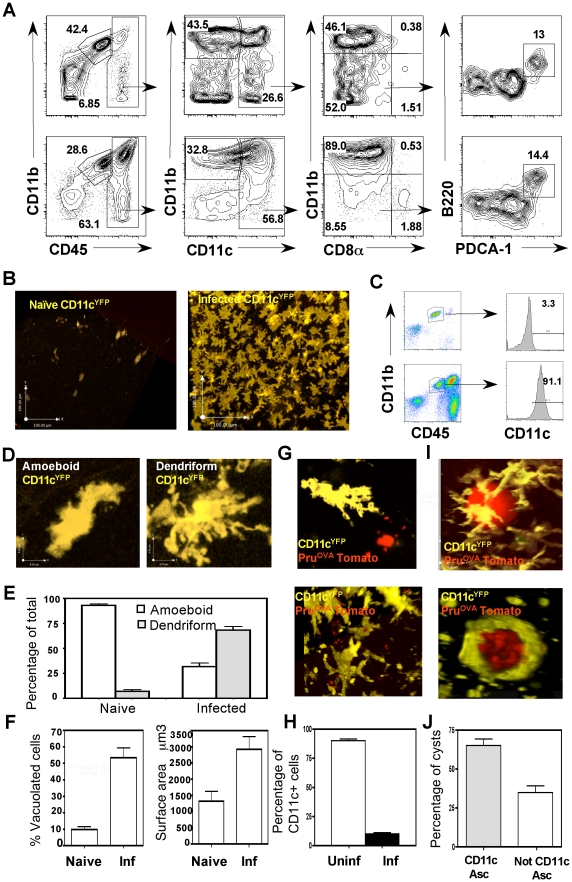
Phenotypic and behavioral characterization of CD11c^+^ cells within the brain. **A**) The various APC populations found within CD3^−^CD19^−^NK1.1^−^ fraction of BMNCs isolated from naive (top) or infected (bottom) mice. The first set of plots shows the relative proportions of microglia (CD45^int^ CD11b^+^) and the other APCs of haematopoietic origin (CD45^hi^). The CD45^hi^ population consists primarily of DCs (CD45^hi^CD11c^+^) and CD11b^+^ cells (macrophages/monocytes). The DC fraction was further subseted by staining for CD8, CD11b, PDCA and B220 to identify the myeloid (CD11b^+^CD11c^+^), lymphoid (CD11c^+^CD8^+^) and plasmacytoid (CD11c^+^CD11b^−^B220^+^PDCA^+^) DCs. **B**) 30 µm z stacks of brain slices from naive and infected CD11c^YFP^ mice (D 21-post infection) imaged by 2-photon microscopy. **C)** The expression of CD11c on the microglia (CD45^int^CD11b^+^) from naive (top) or infected (bottom) mice. **D**) The two prominent types of morphologies (amoeboid and dendriform) observed amongst the CD11c^YFP^ cells in the brain. **E**) The relative proportion of the amoeboid and dendriform cells (expressed as a percentage of the total CD11c^YFP^ cells) analyzed from 30 µm z stacks of brain slices from naive and infected CD11c^YFP^ mice. **F**) **Left**: Average number of vacuolated cells from multiple z stacks of brain slices from naive and infected CD11c^YFP^ mice. Cells with greater than 2 vacuoles were included in the analysis. **Right**: The average surface area of CD11c^YFP^ cells that was calculated from multiple z stacks (30 µm) of brain slices from naive and infected mice. **G**) CD11c^YFP^ cells seen in association with a reactivating cyst (top) and tachyzoite infected CD11c^YFP^ cells (bottom). **H**) The percentage of CD11c^+^ cells that were either uninfected (open bars) or infected (black bars) amongst BMNC isolated from Pru^OVA^ tomato infected mice. **I**) CD11c^YFP^ cells in close association with parasite cysts (top panel). A cyst (showing individual bradyzoites) wrapped in YFP^+^ cells (bottom). **J**) The percentage of cysts observed across multiple imaging regions, that were either associated with or not associated with CD11c^YFP^ cells.

TE is associated with increased numbers of CD11c^+^ cells within the brain [17], however little is known about the subset composition, phenotype and behavior of these cells *in vivo*. Therefore, the APC populations within the brain during TE were characterized by flow-cytometry. In uninfected mice there were minimal numbers of immune cells present in the brain, and microglia (CD45^int^ CD11b^+^ cells) were the dominant immune population (Figure 1A top). During TE, in addition to the microglia, there is an increase in the number of APCs (CD3^−^CD19^−^NK1.1^−^) of haematopoietic origin (CD45^hi^) (Figure 1A bottom). These include macrophages (CD11c^−^CD11b^+^) and DCs (CD11c^+^). The myeloid DCs (CD11b^+^CD11c^+^) form the largest subset (about 80–90% of the CD11c^+^ fraction), followed by the lymphoid (CD8^+^CD11c^+^) and plasmacytoid (PDCA^+^ B220^+^) subsets (each constitute <5% of the CD11c^+^ population). All of these populations were activated based on their expression of high levels of markers such as MHC I, MHC II, CD80 and CD86 (Figure S1).

To directly visualize CD11c^+^ cells within the brain during TE, CD11c^YFP^ mice were used. In these reporters, few YFP^+^ cells were identified within the brain parenchyma in naïve mice (Figure 1B). During TE, consistent with the flow-cytometric data, there was an increase in the number of CD11c^YFP^ cells (Figure 1B), which formed an extensive network within the brain parenchyma. Analysis of YFP^+^ cells revealed that they consisted of DCs (CD11c^hi^CD45^hi^) and microglia (CD11b^hi^CD45^int^), consistent with previous reports that microglia can up-regulate CD11c during inflammation [30]–[32] (Figure 1C).

*In situ* imaging by multiphoton microscopy using the CD11c^YFP^ mice revealed two distinct morphological phenotypes: amoeboid and dendriform cells (Figure 1D). In the naive brain the CD11c^+^ cells exhibited an amoeboid structure whereas during infection there was an increased proportion of dendriform CD11c^+^ cells (Figure 1E). In comparison to the cells seen in the naïve brain, during TE there was marked increase in the average surface area of the CD11c^+^ cells, (Figure 1F right) and an increased proportion of CD11c^+^ cells that displayed a vacuolated phenotype (Figure 1F left). Using fluorescent parasites (Pru^OVA^ Tomato), infected cells could be identified (Figure 1G), however a large proportion of the vacuolated cells were not associated with parasites. The presence of vacuoles in the absence of identifiable parasites has been noted previously both with DCs in the lymph node and astrocytes in the brain during *T. gondii* infection [8], [14] and is indicative of an activated phenotype. The proportion of CD11c^+^ cells (DCs and microglia) that were infected with parasites was less than 10% (Figure 1H). CD11c^+^ cells were also observed associated with parasite cysts within the CNS (Figure 1I, Video S1), with about 60–70% of the cysts intimately associated with CD11c^+^ cells (Figure 1J, Video S2). Together, these studies show that TE results in a significant increase in CD11c^+^ cells composed of a mixed population of activated microglia and dendritic cells, which exhibit distinct morphologies and behaviors.

### DC-T cell interactions during TE

**Figure 2:**
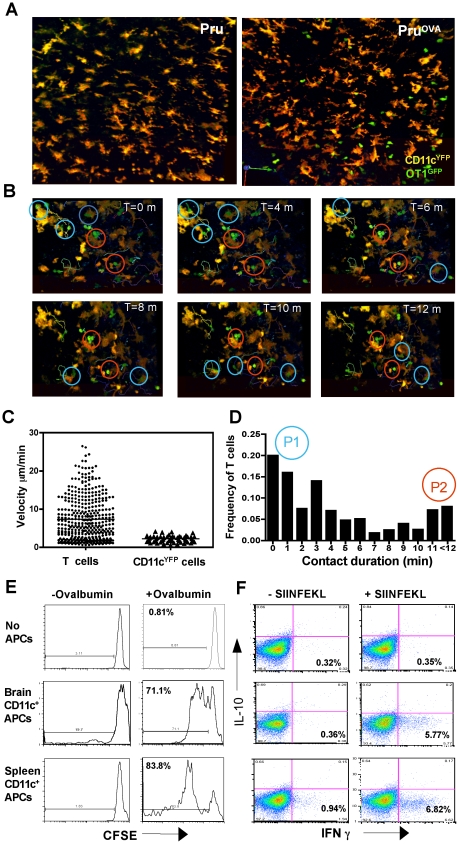
CNS resident CD11c^+^ cells are efficient antigen presenting cells. **A**) Representative z stacks (30 µm) of brain slices from CD11c^YFP^ mice that received OT1^GFP^ cells and were infected with Pru (left) or Pru^OVA^ (right) 3 weeks prior. **B**) Representative time series showing short term (blue circles) and sustained (red circles) interactions between OT1^GFP^ and CD11c^YFP^ cells. **C**) The average migration velocities of OT1^GFP^ T cells and CD11c^YFP^ obtained by manual tracking using Volocity. **D**) The frequency of OT1^GFP^ cells interacting with CD11c^YFP^ cells and the duration of these interactions is shown on the y-axis. The imaging sessions typically lasted between 12–15 minutes. **E**) CFSE dilution profile of naïve OT1 cells that were co-cultured (3 days) with CD11c^+^ cells purified from either spleen or brain (of chronically infected mice). **F**) The ability of the proliferated cells to produce the cytokines IFN-γ and IL-10, during re-stimulation (6 hours) *in vitro* in the presence of brefeldin-A, with/without antigenic peptide (SIINFEKL).

Since CNS resident DCs can have immuno-stimulatory or tolerogenic properties, studies were performed to assess whether the CD11c^+^ cells present in the brain during TE can present antigen to T cells. Naïve OT1^GFP^ T cells were adoptively transferred into CD11c^YFP^ mice followed by infection with Pru or Pru^ova^ parasites, to determine whether antigen specific T cells interacted with DCs *in vivo*. In CD11c^YFP^ mice infected with parental strain of the parasite (Pru) there was minimal infiltration of OT1^GFP^ cells into the brain (Figure 2A), but during infection with Pru^OVA^, significant numbers of T cells were present within the brain (Figure 2A, Video S3). The OT1^GFP^ cells interacted with the CD11c^YFP^ cells and made sustained contacts (highlighted with red circles) or short-lived contacts (highlighted with blue circles) as seen in the representative time series (Figure 2B). Further analysis revealed that the T cells showed a range in their motilities (average velocity ∼10 µm/min) with highly motile cells, cells that paused briefly and cells that were rounded up and stationary for much of the imaging period (10–15 minutes). In contrast, the CD11c^+^ cells showed minimal motility (average <2 µm/min) (Figure 2C left). The duration of the interactions between the T cells and DCs was calculated and the frequency of T cells exhibiting different contact durations was determined (Figure 2D). Majority of the T-DC interactions observed were between uninfected DCs and T cells, which is consistent with the data that only a small percentage of CD11c^+^ cells are infected. (Figure 1H). Analysis of the interaction times revealed two populations: a larger population (P1) of motile T cells that interacted with CD11c^+^ cells for shorter durations (less than 4 minutes) and a smaller subset (P2) of more rounded T cells that were engaged in prolonged interactions with DCs (Figure 2D). The prolonged interactions were however not restricted to the infected DCs and could thus be indicative of antigen presentation or target cell lysis.

Since CNS resident DCs have been proposed to generate tolerogenic T cell responses [21], [33], [34], the ability of DCs present in the CNS during TE to prime naïve antigen specific T cells was evaluated. CD11c^+^ cells were isolated from either the brain or spleens of infected mice, incubated with ovalbumin and co-cultured with naïve CFSE labeled OT1 T cells. The OT1 T cells that were primed by the CD11c^+^ cells from the brain proliferated (as indicated by dilution of CFSE), albeit at a slightly lower rate compared to the T cells that were primed by splenic DCs (Figure 2E). The CD8^+^ T cells primed under the two different conditions (splenic or brain CD11c^+^ cells) exhibited a similar pattern of cytokine secretion and produced IFN-γ but not IL-10 (Figure 2F). Control experiments were also performed to compare DCs isolated from the spleens of naive and infected mice and these studies did not reveal any significant differences in their ability to stimulate OT1 T cells (data not shown). Together, these data indicate that the CD11c^+^ cells found during chronic toxoplasmosis are capable of processing and presenting antigen to CD8^+^ T cells and do not skew naïve T cells to a tolerogenic phenotype.

### Chemokine receptor profile of CNS resident CD11c^+^ cells

**Figure 3:**
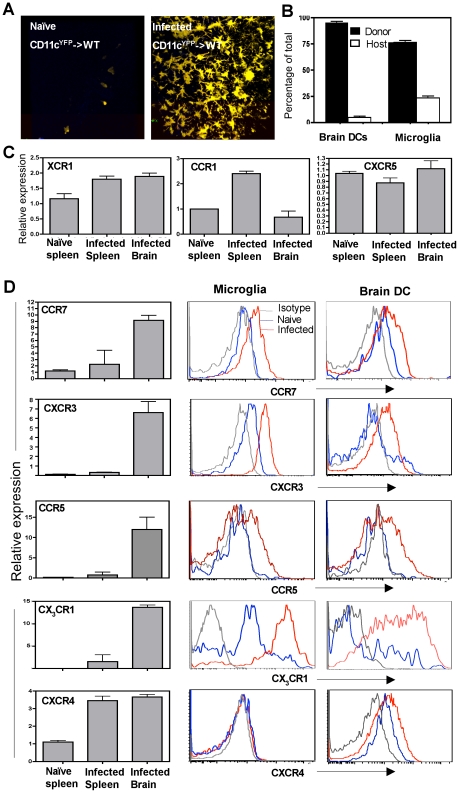
CNS resident CD11c^+^ cells express a distinct chemokine receptor expression profile. **A**) Brain slices from naïve (left) or infected (right) CD11c^YFP^->WT bone marrow chimeras imaged by 2 photon microscopy **B**) The proportion of donor (CD45.2) and host cells (CD45.1) amongst the DC and microglial compartment of the brain from infected CD45.2->CD45.1 radiation bone marrow chimeras. **C**) The expression of the chemokine receptors XCR1, CCR1 and CXCR5 on CD11c^+^ cells purified from the brain or spleen of infected mice measured by real time PCR, normalized to the expression of endogenous house-keeping gene HPRT and expressed as fold increase over control (splenic DCs from naive mice). **D**) **Left:** The relative expression of chemokine receptors CCR7, CXCR3, CCR5, CX_3_CR1 and CXCR4 measured by real time PCR from CD11c^+^ cells isolated from the brain and spleens of naïve and infected mice. **Right:** Flow cytometric analysis of the same chemokine receptors on DCs and microglia from naive (blue) or infected mice (red) compared to the isotype control (grey).

The source of the CD11c^+^ cells found within the CNS during TE is not clear and several alternate mechanisms have been proposed for their recruitment and retention during neuro-inflammation. In order to identify whether CD11c^+^ cells present in the brain during TE were derived from haematopoietic precursors, bone marrow cells (depleted of mature T cells) from CD11c^YFP^ transgenic mice were used to reconstitute lethally irradiated WT recipient mice. In such CD11c^YFP^->WT chimeras very few CD11c^YFP^ cells (bone marrow derived) could be found within the brain in the naïve mice (Figure 3A). However, during chronic TE, significant accumulation of CD11c^YFP^ cells was observed within the brain (Figure 3A). Radiation chimeras were also generated using congenic mice, which differed in the CD45 allotype (CD45.1->CD45.2) to distinguish donor and recipient cells. These studies revealed that greater than 95% of the DCs and approximately 70-75% of the microglia found within the brain during TE were of donor origin (Figure 3B) indicating that the microglia and DCs present within the brain during chronic TE are largely bone marrow derived.

In order to understand the factors that influence the recruitment of DCs to the brain during TE, the levels of chemokine receptor mRNA and surface expression on CD11c^+^ cells within the brain during infection were compared to those in the periphery. The number of CD11c^+^ cells that could be isolated and purified from an uninfected brain was minimal and precluded analysis. Nevertheless, there were significant differences in the levels of chemokine receptor mRNA expression between CD11c^+^ cells isolated from the brains of infected mice compared to splenic populations. While chemokine receptors such as XCR1 and CXCR5 were not altered, others such as CCR1 were lower on the CNS resident DCs compared to splenic DCs (Figure 3C). However, CD11c^+^ cells from the brain expressed increased levels of transcripts for CCR7, CXCR3, CCR5 and CX_3_CR1 (Figure 3D left) and flow cytometric analysis showed that both microglia and DCs upregulated these chemokine receptors. In contrast, CXCR4 was expressed only on the DCs and not on microglia during infection. These studies identify multiple chemokine receptors that represent candidates to mediate DC migration and retention within the brain.

### Adoptive transfer system for DC trafficking

To understand the factors that control the recruitment and retention of DCs during TE, an adoptive transfer system was developed to quantify the migration of DCs into this site. Initial experiments using either immature or mature bone marrow DCs showed limited migration of this DC subset into the CNS (data not shown). As an alternate approach, CD11c^+^ cells that were expanded *in vivo* with FLT3L expressing tumor cells and subsequently purified using CD11c^+^ MACS beads, were used as a source of DCs [35]. Morphologically these cells resemble DCs (Figure S2A) and flow cytometric analysis of these cells showed that they are CD11c^+^, lineage negative (CD3^−^CD19^−^NK1.1^−^), MHC class II^+^ and the proportion of various DC subsets is comparable to those found in naïve spleens (CD11b^+^CD11c^+^, CD11b^−^CD8^+^) as seen previously [35], [36]. The *in vivo* expanded DCs showed a chemokine receptor pattern typical of mature splenic DCs and express CCR7, CXCR3, CCR5 and CX_3_CR1 (Figure S2A).

**Figure 4:**
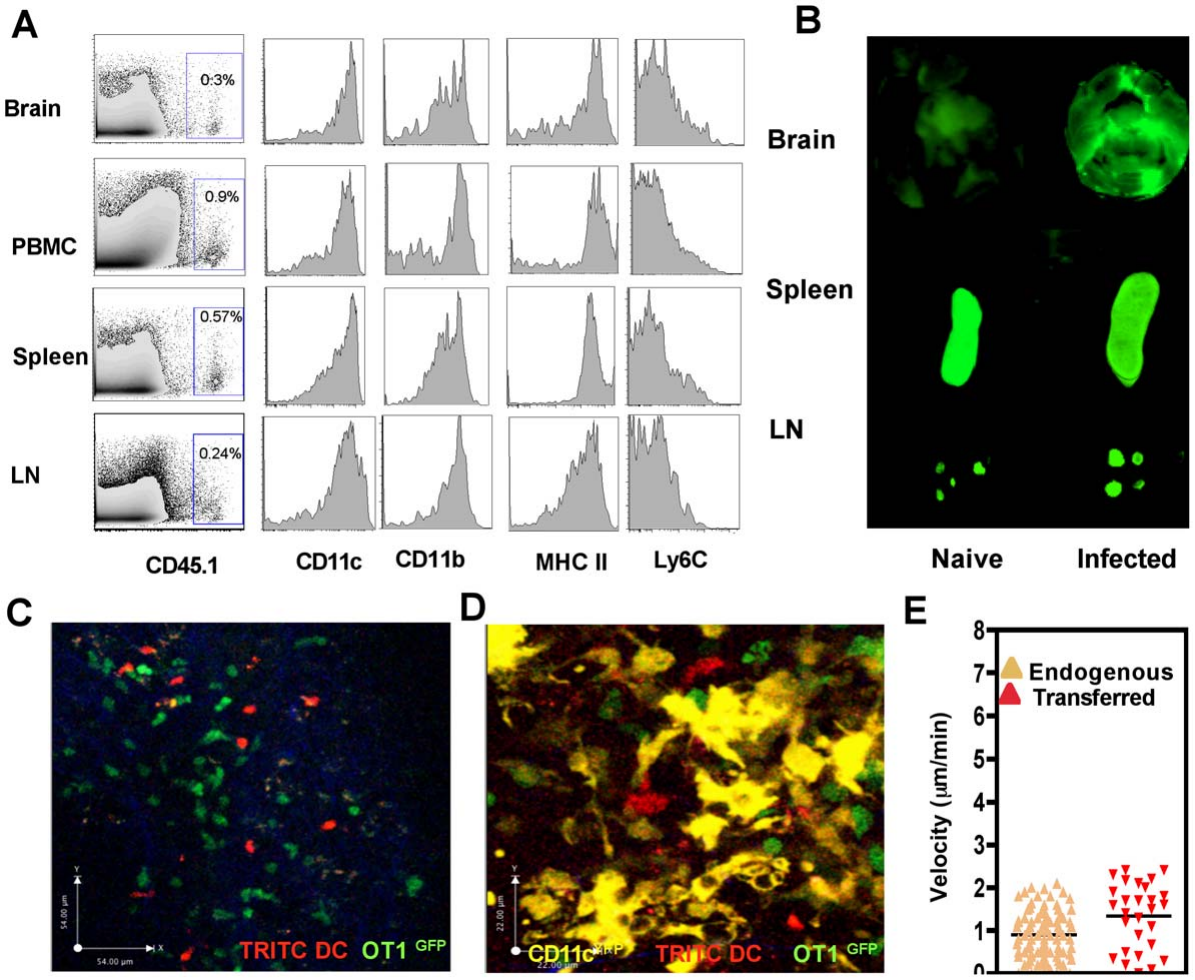
Adoptively transferred DCs migrate from the periphery into the inflamed brain. **A**) Transferred DCs (CD45.1) recovered from the brain, spleen, lymph nodes and peripheral blood of recipient animals 18–24 hours later. The expression of CD11c, CD11b, MHCII, and Ly6C on the CD45.1^+^ fraction (transferred population) is shown in the panels on the right. **B**) Whole organ imaging using a Licor Odyssey imager of the brain, spleen and cervical lymph nodes from naïve or infected mice that received DCs loaded with near infrared dye labeled polymersomes. **C**) Snapshot showing TRITC labeled DCs and OT1^GFP^ T cells in a brain slice from a Pru^OVA^ infected mouse. **D**) Snapshot showing transferred DCs (red), OT1^GFP^ T and endogenous CD11c^YFP^ cells in the infected brain. **E**) The average migration velocity of transferred DCs (red triangles) and endogenous CD11c^YFP^ cells (yellow triangles).

To determine if these DCs could traffic into the brain during chronic TE, they were transferred iv into naïve or infected mice and were tracked by 1) using congenic markers (CD45.1) 2) loading DCs with NIR^−^polymersomes or 3) labeling DCs with dyes such as TRITC (Figure S2B). In the first example, using the congenic marker CD45.1, transferred DCs could be tracked within the brain of infected recipients by flow cytometry 18–24 hours post transfer (Figure 4A). Analysis of various markers including CD11c, CD11b, MHCII and Ly6C indicated that the DCs that migrated to the brain were representative of the transferred population and not significantly different from those found in the other compartments such as the blood, lymph nodes and spleen (Figure 4A). In a different approach to track the transferred population, DCs that phagocytosed polymersomes (containing near infrared dyes) were transferred into naïve or infected mice and 18–24 hours later near infrared imaging of whole organs was performed (Figure 4B). In naïve mice, fewer DCs migrated to the brain and these cells were primarily within the spleen and lymph nodes. In contrast, in infected mice significant numbers of polymersome loaded DCs could be detected within the brain (Figure 4B). DCs labeled with TRITC could similarly be tracked within the brain using multi photon microscopy and the transferred DCs could be co-imaged with OT1^GFP^ cells (Figure 4C, Video S4) and endogenous CD11c^YFP^ cells (Figure 4D, Video S5). Morphologically the transferred DCs exhibited lower average surface area and had a slightly higher velocity compared to endogenous DCs (Figure 4E, Video S5). However, these cells exhibited a surface phenotype typical of DCs (CD11c^+^, CD11b^+^, MHC class II^+^, Ly6C^−^) as seen in Figure 4A. In other experiments transferred monocytes were similarly shown to traffic to the brain but exhibited a distinct phenotype (CD11c^−^, Ly6C^+^, CD11b^+^, CD115^+^) compared to the DCs (Figure S3). These approaches validate a model in which the entry of mature DCs from the circulation to the brain during chronic infection can be quantified and these populations can be visualized.

### The migration of DCs into the brain is pertussis toxin sensitive

**Figure 5:**
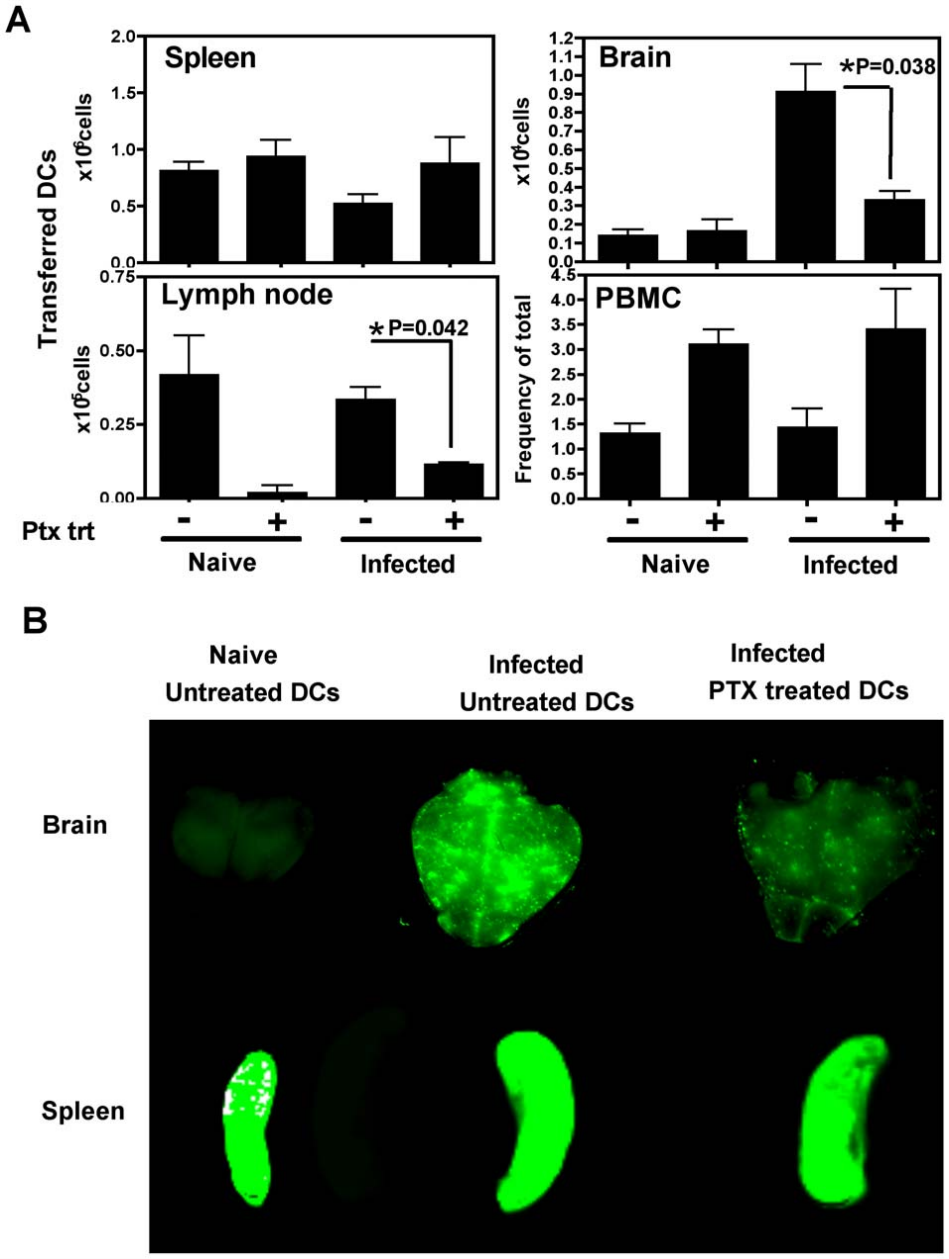
Migration of adoptively transferred DCs into the brain is a pertussis toxin sensitive process. **A**) Total numbers of PTX treated or control DCs (derived from flow-cytometric analysis of the transferred population) within the spleen, brain, lymph node or blood (PBMC) of recipient mice 18–24 h after transfer. **B**) Whole organ near infrared imaging of brain and spleen from infected mice that either received control or PTX treated DCs. Naïve mice that received control DCs is also included. The DCs were loaded with polymersomes containing near infrared dyes to track them.

To determine whether the migration of DCs into the brain was dependent on chemokine receptor mediated signaling, the effect of pertussis toxin (PTX), an inhibitor of Gα_i_ coupled chemokine receptors, was tested. In these studies, DCs were pretreated with PTX *in vitro* for three hours, washed and transferred into naive or *T. gondii* infected mice (day 21 post infection), and analyzed 18–24 hours post-transfer. Flow cytometric analysis revealed that significantly lower numbers of PTX treated DCs accumulated within the brains of infected mice compared to control DCs (Figure 5A). The numbers of DCs in the spleen was not altered by the PTX treatment, however their migration into the lymph nodes was also PTX sensitive. The peripheral blood showed an increase in the number of DCs in the PTX treated group, suggesting that the cells that are not recruited in the tissue compartments remain in the blood. Similar studies using polymersome loaded DCs and whole animal imaging revealed that the accumulation of PTX treated DCs was much lower than untreated DCs within the infected brain (Figure 5B). These data demonstrate that the accumulation of adoptively transferred DCs in the brain during TE is a pertussis toxin sensitive process.

### Chemokine receptors involved in DC trafficking

**Figure 6:**
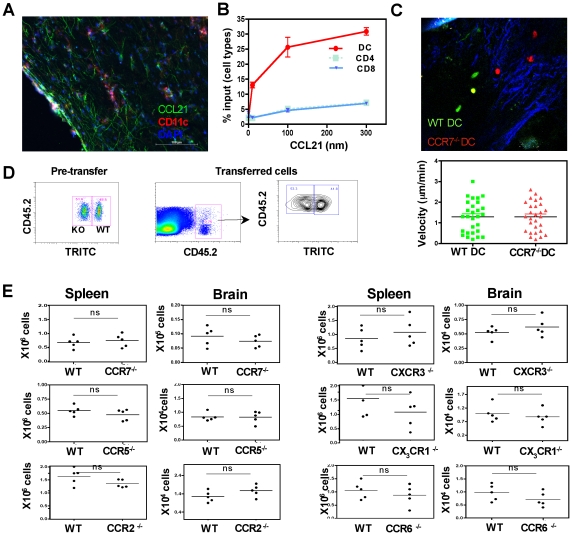
Migration of transferred DCs to the brain is not dependent on CCR7, CCR5, CCR2, CXCR3, CX_3_CR1 or CCR6. **A**) Representative immuno-histochemical staining for CCL21, CD11c and DAPI on 6 µm sections of a chronically infected brain. **B**) The proportion of different cellular subsets (DCs, CD4^+^ and CD8^+^ T cells) from BMNCs, isolated from infected brains that migrate in response to graded doses of the chemokine CCL21 in an *in vitro* chemotaxis assay. **C**) **Top**: Snapshot of a 30 µm z stack from an infected brain imaged using 2P microscopy showing WT and CCR7^−/−^ DCs. Analysis of several such images did not show any significant differences in the localization of the WT and CCR7^−/−^ DCs. **Bottom**: The average velocity of the WT and CCR7^−/−^ DCs. **D**) **Left**: Pre-transfer donor population (CD45.2^+^) that consists of an equal mixture of WT (TRITC^−^) and CCR7^−/−^ (TRITC^+^) cells. **Right**: Donor cells recovered from the spleen of CD45.1^+^ recipient mice where the two different DC populations can be identified. **E**) The total numbers of CCR7^−/−^, CCR5^−/−^ CCR2^−/−^, CXCR3^−/−^, CX_3_CR1^−/−^, CCR6^−/−^ recovered from the spleen and brains in a competitive DC transfer system. The differences between the WT and the various CCR deficient DCs that accumulated in the brain and spleen were not significant (p>0.05 in all cases as measured by student's *t* test).

Since the migration of transferred DCs into the brain was blocked by PTX, the role of individual chemokine receptors in DC migration was tested. Since CCR7 was expressed at high levels on brain DCs, the expression of the chemokine CCL21 (ligand for CCR7) was elevated during infection (Figure 6A), and CCR7 on the brain DCs was functional in *in vitro* chemotaxis studies (Figure 6B), this receptor was first targeted. To directly test the role of CCR7 in the trafficking of DCs into the brain, the transfer system described above was modified to allow co-transfer of equal numbers of CFSE labeled WT and TRITC labeled CCR7^−/−^ DCs. Real time imaging studies indicated that both WT and CCR7^−/−^ DCs could be found in equivalent frequencies within the brain parenchyma (Figure 6C top) and there was no significant difference in their average migration velocity (Figure 6C bottom).

To track these cell populations by flow cytometry CD45.2^+^ WT and CD45.2^+^ TRITC^+^ CCR7^−/−^ were transferred into infected CD45.1^+^ mice thus enabling identification of the WT and CCR7^−/−^ DCs within the same recipients (Figure 6D). However, the absence of CCR7 did not significantly affect the accumulation of DCs in the brain (Figure 6E). The role of other chemokine receptors that were up regulated on the CNS DCs including CCR5, CXCR3, CX_3_CR1, CCR2 and CCR6 were examined using the respective chemokine receptor deficient mice in this competitive transfer model. In these studies the absence of any one of these receptors did not affect the ability of DCs to migrate into the infected brain (Figure 6E), suggesting the cooperative action of multiple chemokines in this process or the role of an as yet untested chemokine receptor/s.

### Migration of DCs into the brain is LFA-1 dependent

**Figure 7:**
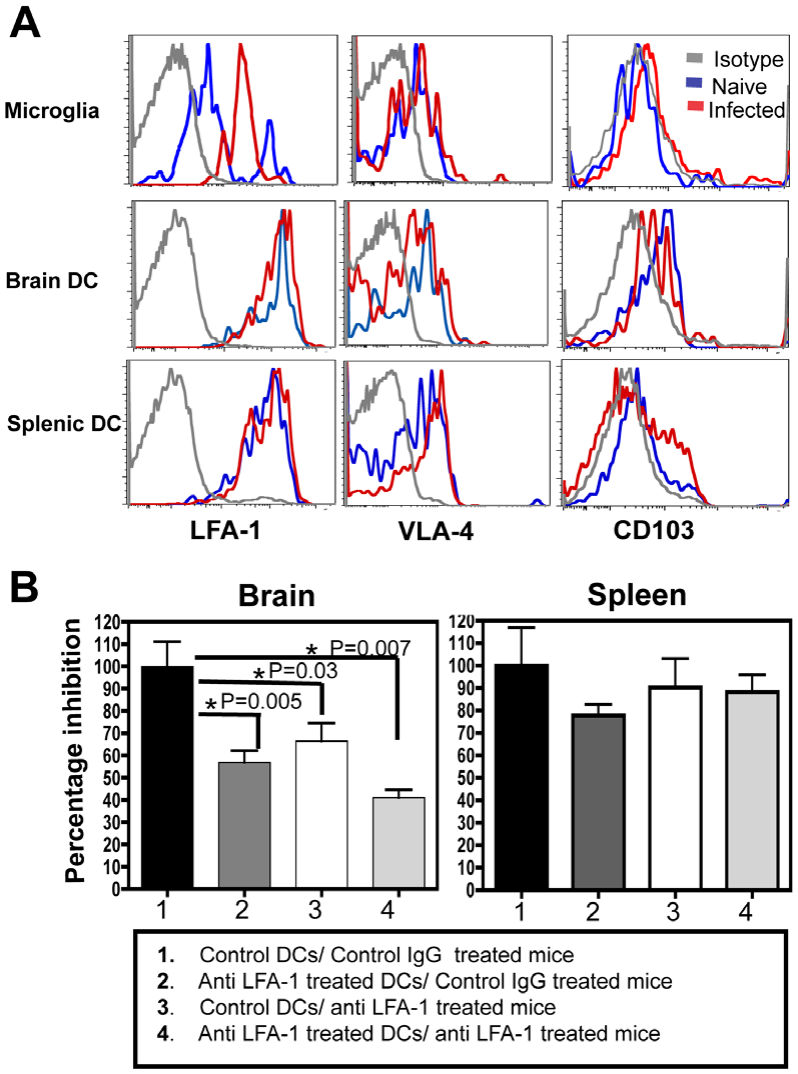
Migration of DCs into the brain is LFA-1 dependent. **A**) Flowcytometric analysis showing expression of LFA-1, VLA-4 and CD103 on the microglia, brain DCs and splenic DCs of naïve (blue) or infected mice (red) compared to the isotype controls (grey). **B**) The percentage inhibition in the accumulation of DCs within the brain or spleen during anti LFA-1 treatment (*in vitro and in vivo*) compared to control conditions. The number of control DCs that were tracked within the brain or spleen of the control mice (Group 1) was normalized to a 100% and the numbers of transferred DCs in the other groups were expressed as a percentage of this control. The treatment conditions for the various groups, is indicated in the legend box within the Figure.

Signaling downstream of the Gα_i_ coupled receptors has been shown to increase the affinity of many integrins particularly LFA-1 and VLA-4, for their respective ligands [37]–[40]. Since our studies showed an effect of pertussis toxin treatment on migration of DCs into the brain, the role of integrins in this process was examined. Previous studies have highlighted the importance of VLA-4 in the migration of T cells into the inflamed CNS [9], [41] however the role of integrins in the migration of DCs to this site is less clear. Flowcytometric analysis of BMNCs revealed that several integrins including LFA-1 (αLβ2) and VLA-4 (α4β1) were expressed by the microglia and DCs, but there was minimal expression of CD103 (αEβ7) by the CD11c^+^ cells from the brain (Figure 7A). Of the various integrins tested LFA-1 was expressed at the highest level by the DCs. While DCs in the brain or spleen did not change their expression of LFA-1 during infection, the microglia showed an upregulation of LFA-1. To test whether high levels of LFA-1 seen on the surface of the DCs had a functional role in their migration to the CNS, the high affinity form of LFA-1 was blocked using monoclonal antibodies in the adoptive transfer model with DCs. DCs were pretreated in culture for 3 hours with anti-CD11a antibody (or control antibody), or alternatively, the mice were treated with the blocking antibodies prior to DC transfer. Both pre-treatment of DCs with anti CD11a antibody and *in vivo* antibody administration, resulted in an inhibition in the ability of the DCs to migrate to the brain (Figure 7B: Group 1 versus Group 2 and 3). Mice that received anti CD11a treated DCs and were additionally treated *in vivo* with the blocking antibody displayed the most pronounced inhibition (Group 1 versus Group 4). Blocking VLA-4 had minimal effect on the migration of DCs into the brain using this adoptive transfer system (Figure S4). Together these data show that the migration of adoptively transferred DCs into the CNS during TE is an LFA-1 dependent process.

## Discussion

Dendritic cells have been identified within the CNS during various parasitic, bacterial, viral infections and autoimmune conditions such as EAE [6], [18], [19], [42], [43]. However, there is no clear consensus about the function of CNS resident DCs, their origin, behavior and migratory patterns. Thus, several studies have highlighted that CNS DCs are inhibitory for adaptive immune responses [21], [22], [33], while others have indicated that they are capable of inducing inflammatory T cell responses [18], [19], [44]. The present studies indicate that the CD11c^+^ cells isolated from the brain during TE are capable of processing and presenting antigen to naïve T cells *in vitro* to generate functional T cell responses. The *in vivo* imaging studies show that while the majority of the T cells interacted transiently with the CD11c^+^ cells, a smaller subset of T cells engaged in prolonged interactions, could be identified which is consistent with the T cell-APC contacts observed recently by Robey and colleagues in the brain [45]. Our studies also revealed that only a small proportion of the DCs that were interacting with the T cells were actively infected and the prolonged interactions were not restricted to the infected cells, which suggests the involvement of both direct and cross presentation pathways in the activation of the CD8^+^ T cells. These data are in support of a model in which DCs and microglia provide local tonic signals to maintain the effector functions of the intracerebral T cells. In addition, the imaging studies have revealed the close association between parasite cysts and CD11c^+^ cells. Neurons are considered to be the major cell type that houses cysts [46], and based on immunohistology the regions around the cyst were thought to be largely quiescent. However, the current studies reveal that the microglia and DCs are intimately associated with the parasite cysts with their dendrites and cell bodies tightly wrapped around the cysts, suggesting possible innate sensing of this stage of the parasite.

Several alternate models have been proposed for the origin of DCs within the brain. Our studies using radiation bone marrow chimeras shows that the majority of the CD11c^+^ cells (>95%) that expand in the CNS during TE are bone marrow derived. This could represent constant recruitment of DCs from the periphery during infection or a local expansion of bone marrow precursor cells that initially seeded the CNS. Recent studies by Merad and colleagues has shown that the microglia are derived from primitive myeloid progenitors and not from post natal haematopoietic precursors [47], [48]. However several other studies indicate that damage and inflammation within the CNS can result in greater proportions of haematopoietic precursor derived microglia [49], [50]. In the present studies, the majority of the microglia found during TE are bone marrow derived. Interestingly the CD45^int^ microglia are derived from the CD45^hi^ precursors, however whether the down-regulation of CD45 occurs within an intermediate precursor population or on the cells that are resident within the brain for prolonged periods, is not completely clear.

The adoptive transfer studies suggest that migration of DCs from peripheral circulation is one of the mechanisms that contribute to the accumulation of the CD11c^+^ cells within the brain during TE. Migration of monocytes and their conversion to DCs is another proposed mechanism that contributes to the DC populations within the inflamed CNS and our preliminary studies using purified monocytes indicate that they can migrate to the brain during TE (Figure S3). While conversion of the monocytes to DCs was not observed immediately (18-24 hours) after transfer, whether this happens over time, remains to be tested.

Several chemokines and their receptors, including CCR7 and its ligands (CCL19 and CCL21) and the CCL3/CCR5 receptor ligand system have been implicated in the migration of DCs into the CNS, primarily based on EAE studies and clinical data from MS patients [51]-[53]. During chronic TE, several chemokines (CCL19, CCL21, CCL3, CCL5, CXCL9, CXCL10) are up regulated within the CNS [54], [55]. In our studies the adoptively transferred DCs migrated to the brain through a pertussis toxin sensitive mechanism, suggesting that the signaling though the Gα_i_ coupled chemokine receptors was involved in this process. Since the lack of any individual chemokine receptor did not affect the ability of these DCs to migrate to the brain, we conclude that this process is possibly mediated through the synergistic effect of signaling events downstream of multiple receptors [56]. A downstream effect of signaling through Gα_i_ coupled receptors results in changes in the conformation of integrins from low to high affinity forms and has been shown to control the migration of immune cells into lymph nodes [37]–[40]. Recently the integrin VLA-4 has been shown to be involved in the recruitment of immature DCs into the CNS during EAE [34]. In our model, the migration of DCs into the brain was found to be dependent on LFA-1, but not VLA-4. Thus, our current studies are consistent with a model in which signaling events downstream of multiple Gα_i_ coupled chemokine receptors results in changes in the affinity of LFA-1 on the surface of the DCs and influences their recruitment into the inflamed CNS [37]–[40], [56].

Until recently the role of integrins within the CNS, was largely focused on the effects of VLA-4 in the migration of T cells and this forms the basis for the clinical development of a monoclonal antibody (Natalizumab) against α4 integrin to treat MS [57], [58]. However, a subset of patients treated with Natalizumab developed progressive multifocal leukoencepahalopathy, due to reactivation of latent JC polyoma virus infection [59]. Interestingly another monoclonal antibody (Efalizumab) directed against LFA-1, which was developed for the treatment of Psoriasis resulted in a similar complication [60]. These clinical studies indicate the importance of LFA-1 in addition to VLA-4 in the migration of immune effectors into the CNS. The identification of LFA-1 as an important molecule required for the recruitment of APCs into the CNS bears significance for the design of therapeutic targets to control CNS inflammation.

## Materials and Methods

### Mice

WT C57BL/6 mice and CD45.1 congenic mice were purchased from Jackson laboratories (Bar Harbor, ME). OT1^GFP^ mice were obtained by crossing DPE-GFP transgenic mice to OT1 TCR transgenic mice, as described previously [14]. CD11C^YFP^ transgenic mice were obtained from Michel C Nussenzweig [61]. CCR2^−/−,^, CX_3_CR1^−/−^, CCR7^−/−^, CCR5^−/−^, CCR6^−/−^ were purchased from Jackson laboratories. The CXCR3^−/−^ mice were obtained from Andrew Luster (MGH, Boston MA). The various transgenic mice colonies were maintained in a specific pathogen-free (SPF) facility in the Department of Pathobiology at the University of Pennsylvania in conformance with institutional guidelines for animal care.

### Ethics statement

All animal studies were carried out in compliance with the guidelines of the Institutional Animal Care and Use Committee (IACUC) of the University of Pennsylvania and in accordance with the recommendations in the Guide for the Care and Use of Laboratory Animals of the National Institutes of Health. The animal protocol was approved by the Institutional Animal Care and Use Committee (IACUC) of the University of Pennsylvania, Philadelphia PA (Multiple project assurance # A3079-01).

### Parasites

The Prugniaud strain of *Toxoplasma gondii* was maintained as tachyzoites by serial passage through HFF monolayers. Transgenic parasites engineered to express cytoplasmic tdTomato and to secrete ovalbumin protein into the parasitophorous vacuole (described previously (46)) were maintained by antibiotic selection in the cultures. In general, mice were infected ip with the various strains of *T. gondii* at a dose of 10^4^ parasites per mouse.

### Adoptive transfers

#### 1) T cell transfers

T cells were enriched from lymphocytes isolated from the spleen and pooled peripheral lymph nodes of OT1^GFP^ mice using the mouse T cell enrichment columns (R&D systems, Minneapolis, MN) as described previously [14]. 2×10^6^ purified OT1^GFP^ cells were injected into recipient mice iv. The recipient mice were infected with parasites 24-hours later and the immune responses in the brain were typically analyzed on day 21-post infection.

#### 2) Adoptive transfer of DCs

DCs were enriched *in vivo* by injecting WT or chemokine deficient mice, subcutaneously with 3×10^6^ FLT3L secreting b16 tumor cells. 10–14 days later CD11c^+^ DCs were enriched from the spleens by positive selection using CD11c (N418) micro-beads (Miltenyi Biotec). The CDllc^+^ cells were labeled with the fluorescent dye TRITC for tracking them microscopically and by flow cytometry. Alternatively, congenic markers (CD45.1 or CD45.2) were used to track them in the recipients. 5×10^6^ DCs were transferred iv into chronically infected mice D21.

### LFA-1/VLA-4 blocking

To block LFA-1 or VLA-4 *in vitro,* the purified DCs were treated with 30 µg/ml of anti CD11a antibody (Clone M-17/4, BioXcell, West Lebanon, NH) or anti VLA-4 (Clone PS/2, BioXcell, West Lebanon, NH) for 3 hours prior to transfer. For *in vivo* treatments chronically infected mice were treated with two doses of the anti LFA-1 or anti VLA-4 (150 µg each) antibody, one dose prior to transfer of DCs (−6 hours) and one dose at the time of DC transfer. The control antibody used in these cases was Rat IgG. The mice were sacrificed 18–24 hours after transfer of DCs.

### NIR polymersome loading and whole organ imaging

Purified splenic DCs were loaded with Tat-functionalized near-infrared emissive polymersomes, as described previously [62]. The various organs (brain, spleen and lymph nodes) were isolated from the animals 18–24 hours later and fluorescence imaging was performed using an Odyssey infrared imaging system (LI-COR Biosciences, Lincoln, NE).

### Preparation of tissue for live imaging

Mice were sacrificed by CO_2_ asphyxiation and the brain was removed with minimal damage to the tissue. A horizontal slice of the brain (between 1.5–2 mm thick) was cut as described previously [8] and placed in a temperature controlled imaging chamber and the tissue was held in place using a nylon mesh and an organ holder (Warner Instruments, Hamden CT). The chamber was constantly perfused with warm (37°C) oxygenated (95% O_2_/5%CO_2_) media (RPMI+10% FBS) and the temperature was maintained at 37°C using heating elements and a temperature control probe.

### 2-photon microscopy

*Ex vivo* imaging was done using a Leica SP5 2 photon microscope equipped with a picosecond laser (Coherent Chameleon; 720 nm–980 nm) and tunable internal detectors that allow simultaneous detection of emissions of different wavelengths and second harmonic signals (SHG). EGFP, YFP and tdTomato were excited using laser light of 920 nm. Typically, z stacks of a series of x-y planes with a total thickness of 30 µm and step size of 6 µm were captured. The LAS-AF software (Leica) was used for image acquisition. For 4D data sets, images were captured every 25–30 seconds and the imaging depths are as specified in the different images.

### Image analysis

Volocity software (PerkinElmer, Waltham MA) was used to convert the three dimensional image stacks into time series. Single cell tracking was done by a combination of manual tracking and automated tracking and mean migratory velocities were calculated as described previously. For measurement of T cell–DC interactions, the duration of contacts of all the T cells observed in a given filed of view were calculated for the entire imaging period (typically lasting 12–15 minutes). Cellular contacts were determined manually as a lack of space between the interacting cells and the data obtained from an average of 3–5 imaging regions from 3 individual mice per group were used to obtain the frequency of T cells with different durations of contacts with DCs.

### RNA isolation and real time PCR for chemokines

RNA was isolated from CD11c^+^ cells purified using CD11c MACs micro-beads (Miltenyi Biotech, Auburn, CA) from the brain mononuclear cells or splenocytes isolated from chronically infected mice. TRIzol (Invitrogen) and DNase treated total RNA was reverse transcribed using Superscript II (Invitrogen, Carlsbad CA) using standard protocols. Quantitative PCR was performed with customized primer sets for CCR7, CCR5, CX_3_CR1, CXCR4, XCR1, CXCR5 and HPRT (QIAGEN, Valencia CA) using SYBR green PCR assay system and an ABI 7500 instrument (Applied Biosystems, Carlsbad, CA). The values for the various chemokine receptors were normalized to HPRT and displayed as fold induction over naïve splenic DC controls.

### Isolation of cells from various tissues

Brain mononuclear cells were isolated using a standard protocol described previously [8]. Briefly, mice were perfused with cold PBS, the brain was removed, diced, passed through an 18-gauge needle and digested with Collagenase/Dispase and DNAse for 45 minutes at 37°C. The cell suspension was then washed and fractionated on a 30%–60% percoll gradient (Pharmacia) for 20 minutes. The cells in the interface consisted of mononuclear cells, which were washed prior to experiments. Peripheral blood was obtained from mice by cardiac puncture, and the RBCs were separated using a Lympholyte-M gradient (Cedarlane, ON, Canada). Lymphocytes were isolated from spleens and cervical lymph nodes by mechanical homogenization followed by lysis of RBCs (for spleens) using lysis buffer (0.846% NH_4_Cl)**.**

### Flow-cytometric analysis

Freshly isolated cells were stained with the antibodies purchased from eBioscience (San Diego, CA) or BD Biosciences (San Jose, CA). For intracellular staining the cells were stimulated *ex-vivo* for 5 hours in complete media with Brefeldin A, either in the presence or absence of SIINFEKL peptide (1 µg/ml). Following surface staining, the cells were fixed with 4% PFA for 10 minutes at room temperature. The cells were permeabilized using 0.3% saponin in staining buffer. The stained samples were run on a FACSCanto (BD, San Jose, CA) and results were analyzed using FlowJo software (TreeStar Inc., Ashland, OR).

### Statistical analysis

Statistical significance of differences between groups of mice was tested using the student's *t* test or ANOVA/ Kruskal-Wallis test for multiple groups. In all the cases, p<0.05 was considered significant.

## Supporting Information

Figure S1 **The DCs within the brain show an activated phenotype following infection.** The expression of MHC I, MHC II and CD80 on the total CD11c^+^ DC population (extreme left) and the various subsets of DCs (CD11b^+^, CD8^+^, CD11b^−^CD8^−^ and pDCs) from the BMNCs isolated from naïve (grey-filled) or infected (black) mice.
(TIF)

Figure S2 **Phenotype of DCs used for adoptive transfer.** **A**) DIC image and flow-cytometric characterization of the DCs used for the adoptive transfer. **B**) DCs tracked by various techniques 1) loading with polymersomes containing near infrared dyes (left) 2) TRITC labeled (middle) 3) or by flowcytometry (right) using congenic markers (CD45.1) and polymersomes.
(TIF)

Figure S3 **Monocyte transfer.** The adoptively transferred monocytes (CD45.1) recovered from the brain and other tissues 18–24 hours post transfer. The expression of the markers CD11b, Ly6C, CD115 and CD11c on the recovered populations is shown in the right panels. The Monocytes used for the transfers were isolated from the bone marrow and purified a multistep protocol involving depletion of Ly6G^+^ cells followed by enrichment of CD11b^+^ cells.
(TIF)

Figure S4 **VLA-4 is not involved in the migration of DCs into the brain.** The total numbers of control or anti VLA-4 treated DCs recovered from the brain and spleen of recipient mice that were either treated *in vivo* with anti VLA-4 or control antibody.
(TIF)

Video S1 **Interaction between DCs and parasite cysts.** A representative time-lapse sequence (30 µm stack) showing the CD11c^YFP^ cells within the brain scanning the surface of Pru^OVA^ tomato cysts (day 21 post infection). OT1^GFP^ T cells that were transferred prior to infection can also be seen within these regions. Second harmonic structures can be visualized in blue.
(MOV)

Video S2 **Close association between CD11c^YFP^ cells and parasite cysts.** 3D rendering of a 30 µm z stack showing the intimate association between a CD11c^YFP^ cell and a parasite cyst (individual bradyzoites can be seen) within the brain of a Pru^OVA^ Tomato infected mice.
(MOV)

Video S3 **Interactions between CD11c^YFP^ cells and T cells within the brain during TE.** 3D time-lapse sequence of 30 µm stacks of live brain slices from a CD11c^YFP^ transgenic mouse that was adoptively transferred with OT1^GFP^ cells and infected with Pru^OVA^ parasites. The movie shows OT1^GFP^ cells making contacts with CD11c^YFP^ cells, majority of which are brief. However, a smaller population of OT1^GFP^ cells that indulge in long-term interactions with CD11c^YFP^ can also be seen.
(MOV)

Video S4 **Migration of transferred DCs into the brain.** Maximum projection time-lapse sequence of 30 µm stacks showing TRITC labeled transferred DCs (24 hours after transfer of DCs) within the brain of mice that had received OT1^GFP^ T cells and were infected with Pru^OVA^ 3 weeks earlier.
(MOV)

Video S5 **Co-localization of transferred and endogenous DCs within the brain.** Maximum projection time-lapse sequence showing transferred TRITC labeled DCs (24 hours post transfer) within the brain of a CD11c^YFP^ transgenic mouse that had received OT1^GFP^ T cells and were infected with Pru^OVA^ 3 weeks earlier. The transferred DCs, T cells and endogenous CD11c^+^ cells can be visualized within the same regions of the brain.
(MOV)
