# Supplementary figures and images for: Analysis of Behavior and Trafficking of Dendritic Cells within the Brain during Toxoplasmic Encephalitis
Source: PLoS Pathog. 2011 Sep 15;7(9):e1002246. doi: 10.1371/journal.ppat.1002246 (PMC3174247; doi:10.1371/journal.ppat.1002246)

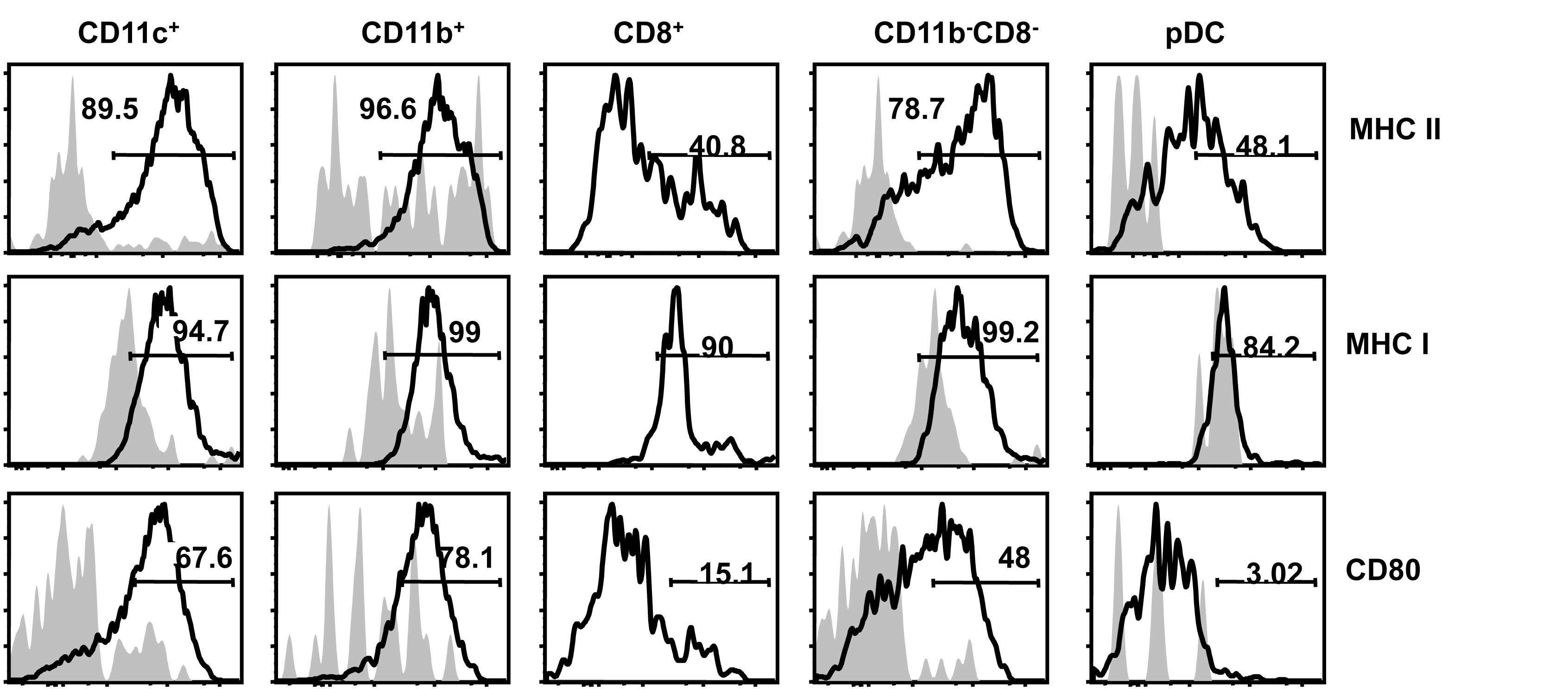

Supplement: Figure S1 — The DCs within the brain show an activated phenotype following infection. The expression of MHC I, MHC II and CD80 on the total CD11c+ DC population (extreme left) and the various subsets of DCs (CD11b+, CD8+, CD11b−CD8− and pDCs) from the BMNCs isolated from naïve (grey-filled) or infected (black) mice. (TIF) [file ppat.1002246.s001.tif]

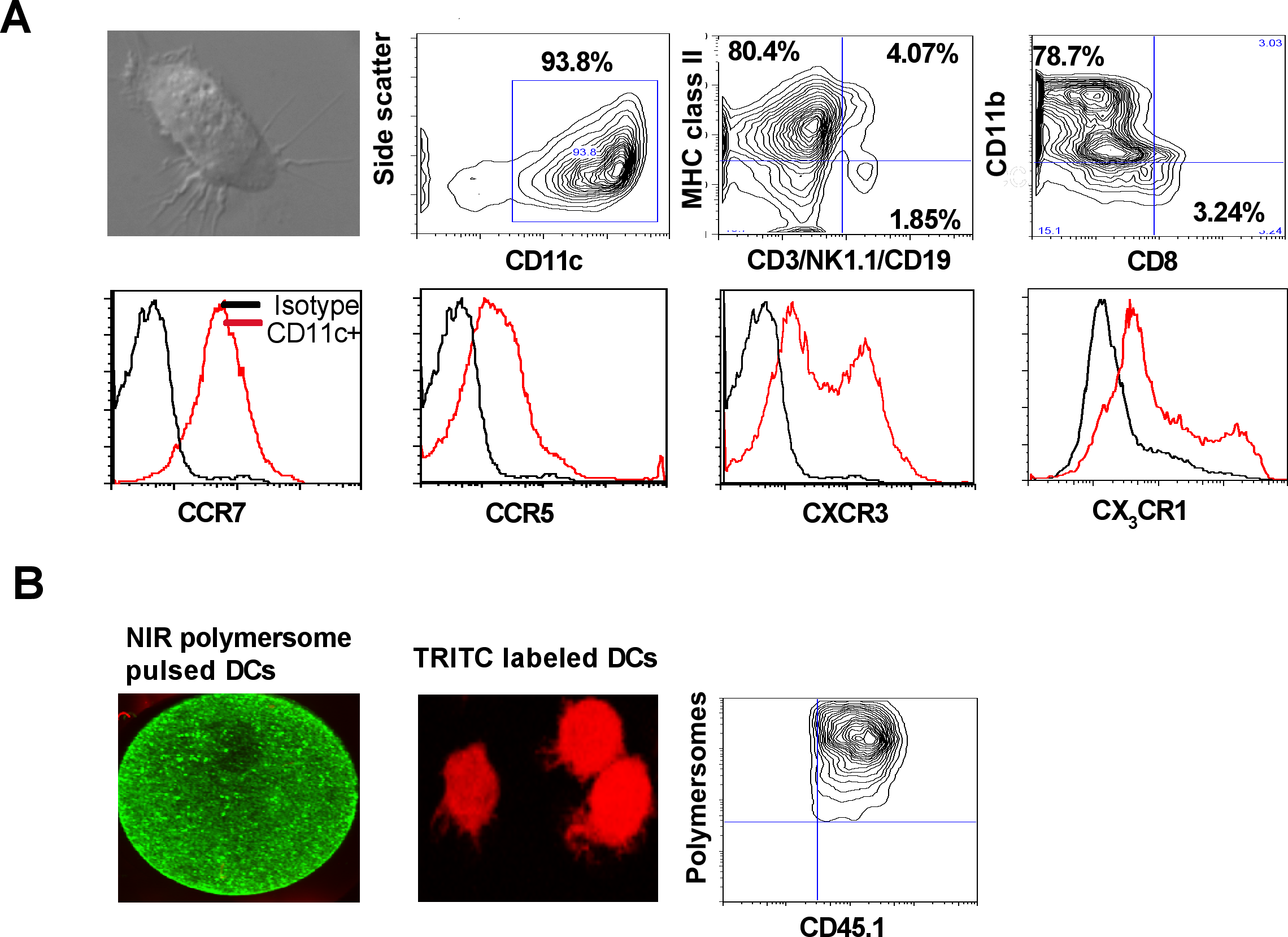

Supplement: Figure S2 — Phenotype of DCs used for adoptive transfer. A) DIC image and flow-cytometric characterization of the DCs used for the adoptive transfer. B) DCs tracked by various techniques 1) loading with polymersomes containing near infrared dyes (left) 2) TRITC labeled (middle) 3) or by flowcytometry (right) using congenic markers (CD45.1) and polymersomes. (TIF) [file ppat.1002246.s002.tif]

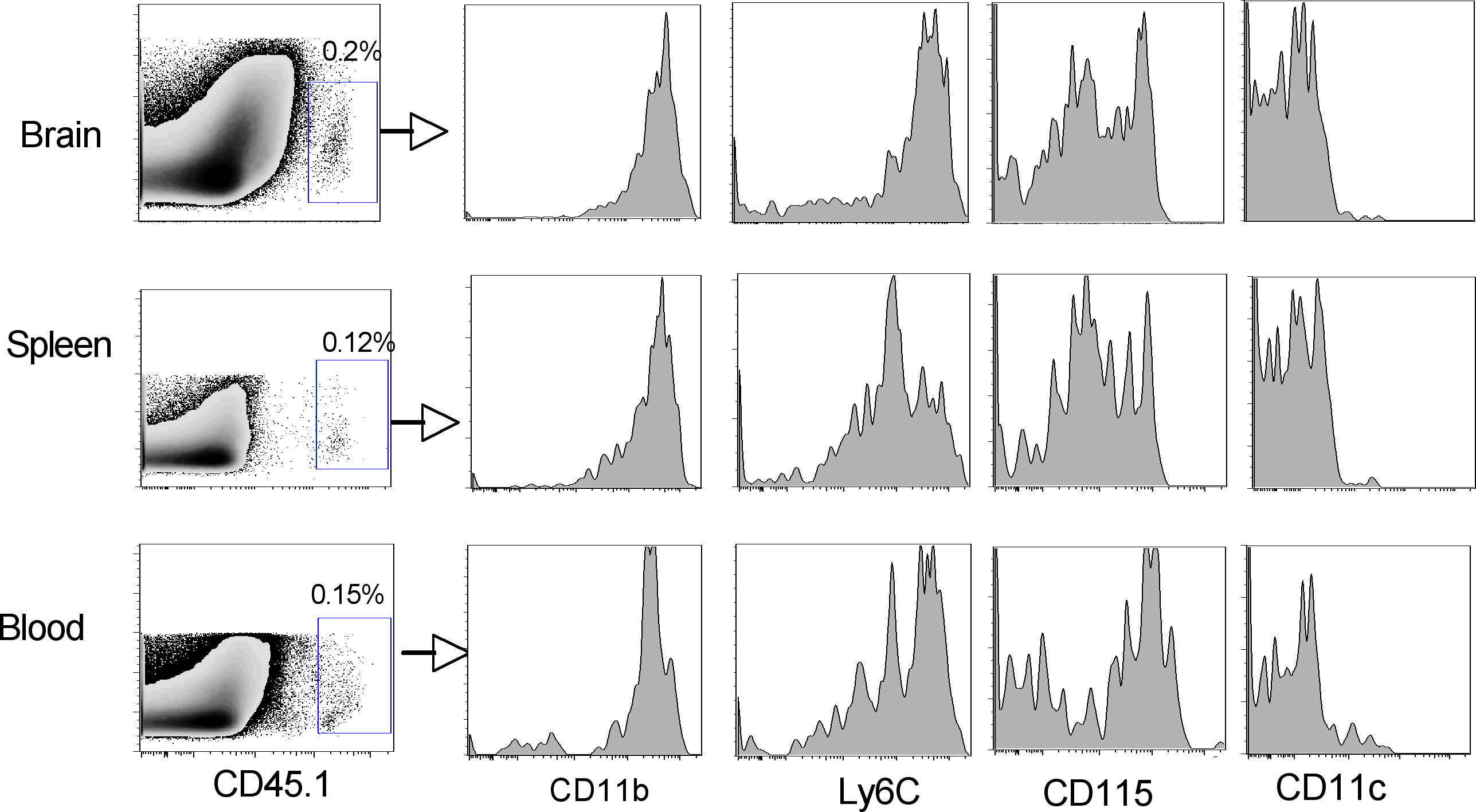

Supplement: Figure S3 — Monocyte transfer. The adoptively transferred monocytes (CD45.1) recovered from the brain and other tissues 18–24 hours post transfer. The expression of the markers CD11b, Ly6C, CD115 and CD11c on the recovered populations is shown in the right panels. The Monocytes used for the transfers were isolated from the bone marrow and purified a multistep protocol involving depletion of Ly6G+ cells followed by enrichment of CD11b+ cells. (TIF) [file ppat.1002246.s003.tif]

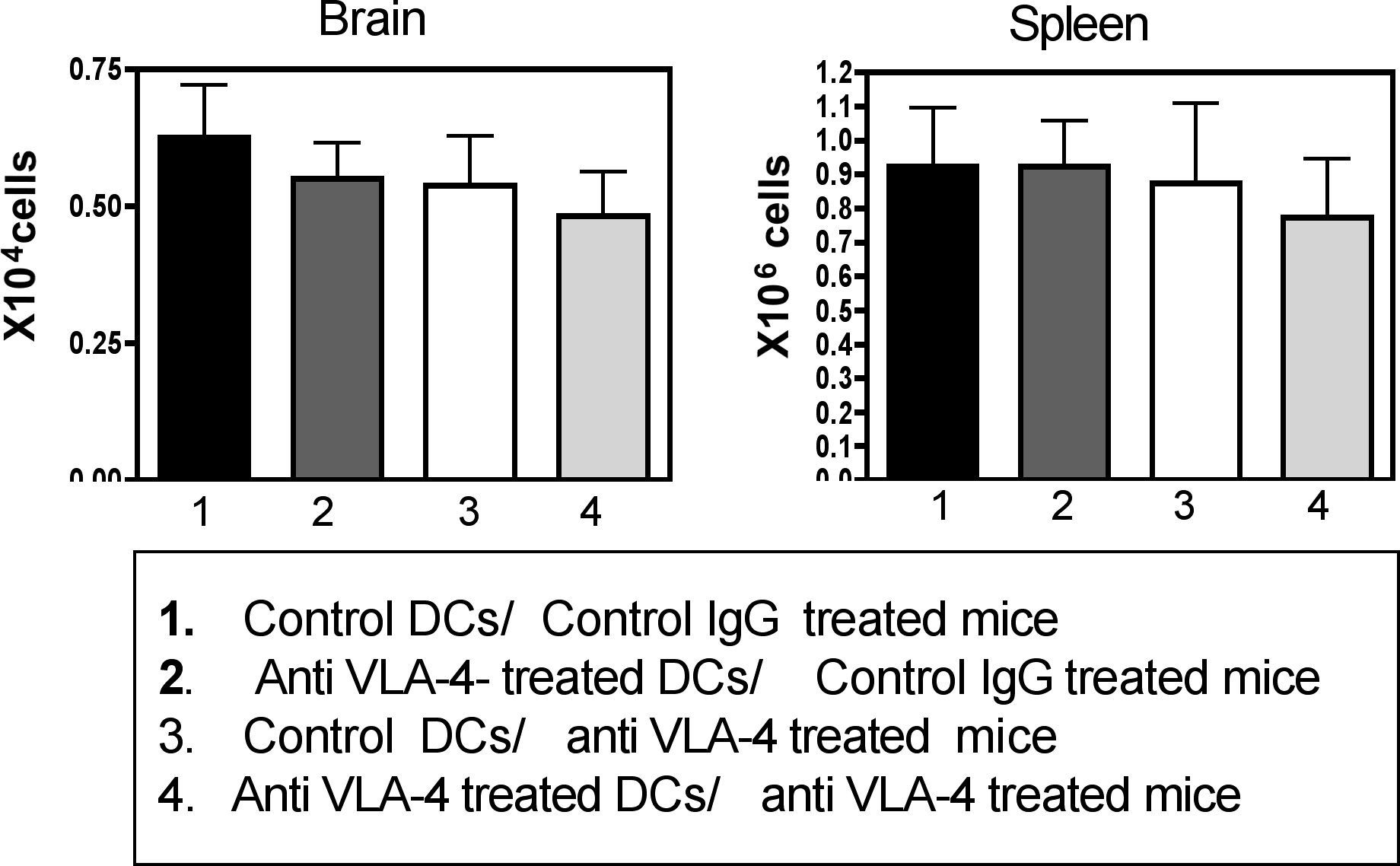

Supplement: Figure S4 — VLA-4 is not involved in the migration of DCs into the brain. The total numbers of control or anti VLA-4 treated DCs recovered from the brain and spleen of recipient mice that were either treated in vivo with anti VLA-4 or control antibody. (TIF) [file ppat.1002246.s004.tif]
